# Cyanobacterial Biocrust on Biomineralized Soil Mitigates Freeze–Thaw Effects and Preserves Structure and Ecological Functions

**DOI:** 10.1007/s00248-024-02389-w

**Published:** 2024-05-10

**Authors:** Keiichi Kimura, Toshiya Okuro

**Affiliations:** Department of Ecosystem Studies, Graduate School of Agricultural and Life Sciences, The University of Tokyo, 1-1-1 Yayoi, Bunkyou-Ku, Tokyo, 113-8657 Japan

**Keywords:** Biological soil crust, Carbohydrate, Freeze–thaw cycle, Land rehabilitation, Microbial-induced carbonate precipitation, Mongolia

## Abstract

Biocrust inoculation and microbially induced carbonate precipitation (MICP) are tools used in restoring degraded arid lands. It remains unclear whether the ecological functions of the two tools persist when these methods are combined and subjected to freeze–thaw (FT) cycles. We hypothesized a synergetic interaction between MICP treatment and biocrust under FT cycles, which would allow both components to retain their ecological functions. We grew cyanobacterial (*Nostoc commune*) biocrusts on bare soil and on MICP (*Sporosarcina pasteurii*)-treated soil, subjecting them to repeated FT cycles simulating the Mongolian climate. Generalized linear modeling revealed that FT cycling did not affect physical structure or related functions but could increase the productivity and reduce the nutrient condition of the crust. The results confirm the high tolerance of MICP-treated soil and biocrust to FT cycling. MICP treatment + biocrust maintained higher total carbohydrate content under FT stress. Our study indicates that biocrust on biomineralized soil has a robust enough structure to endure FT cycling during spring and autumn and to promote restoration of degraded lands.

**Supplementary Information:**

The online version contains supplementary material available at 10.1007/s00248-024-02389-w.

## Introduction

Inoculation with organisms that form biological soil crusts (biocrusts) has become a well-known method for restoring degraded arid lands [1]. Biocrusts often dominate the soil surface in arid and semiarid areas. Their structure consists of sand particles held together by various microorganisms such as cyanobacteria, algae, fungi, lichens, and mosses [2]. Biocrust is known for its many ecosystem functions: high wind erosion tolerance with soil stability enhancement [3], fixation of atmospheric carbon and nitrogen [4], and support of vegetation establishment and succession [5]. Biocrust also seems to have a higher tolerance to environmental stresses, such as extreme soil temperature changes [6], in degraded drylands than plants [7]. Therefore, biocrusts are being investigated for use in the rehabilitation of degraded land [1]. However, several barriers prevent their introduction, notably soil surface instability. Sand-stabilizing techniques such as the use of soil amendments and tackifiers have often been used with biocrust inoculation to assist biocrust colonization [8–11]. Successful biocrust inoculation depends on the combination of biocrust species and the supporting substrate [9].

A possible technique to support the biocrust settlement is microbially induced carbonate precipitation (MICP), a biochemical process induced by ureolytic bacteria. Under appropriate microenvironmental conditions (e.g., pH or Ca^2+^ concentration), ureolytic bacteria perform biomineralization by hydrolyzing urea into ammonia and carbon dioxide [12, 13]. They then precipitate calcium carbonate (CaCO_3_), which coats and bonds sand particles, forming a crust that hardens the soil surface [14]. Microbial biomineralization of sand dunes can improve soil stability and soil moisture content by forming stiff crusts on the surface, strengthening the soil surface against erosion caused by water and wind [15] and contributing to sand fixation [14, 16]. It is, however, still unclear how MICP treatment affects other microorganisms, including cyanobacteria, in the target soil [17, 18] and how long the stabilizing effect continues under field conditions [17].

A critical environmental stress on both biocrust development and the durability of biomineralized soil is the freeze–thaw (FT) cycle [6, 19]. Water that remains in porous solids becomes ice when it freezes, causing mechanical damage and deterioration of crusts [20]. Although some biocrust organisms have high resistance to the FT cycle and extreme temperature changes [6, 21], the FT cycle may change the biocrust’s structure and ecological functions. The extent of this effect on the biocrust depends on both the biocrust’s developmental level and the FT cycle’s intensity [21, 22], but the influence of FT cycles is still unclear on the biocrust applied with soil amendment. Biomineralized soil, on the other hand, can withstand the FT process by an appropriate porosity arrangement [23]. However, high porosity means lower CaCO_3_ precipitation and weaker physical strength, and low porosity inhibits plant seed germination [24]. It is still unclear whether biomineralization treatment can balance the tolerance for FT cycles and the survival of organisms in degraded lands.

The aims of this study were to confirm whether cyanobacterial biocrusts on MICP-treated soil could retain their ecological functions after FT cycles and to confirm the synergy and competition between the two rehabilitation techniques under FT stress. We hypothesized that MICP treatment would be beneficial to biocrust establishment because of its physical strength, even if weakened by FT cycles. Biocrust could also enhance soil physical strength and ecological functions through its incorporation of biomineralized sand particles. To test this hypothesis, we evaluated whether biocrust and biomineralized sandy soil keep their ecological functions after FT cycles.

## Methods

### Experiment Design

We cultured *Nostoc commune* (UTEX B 1621, University of Texas at Austin Culture Collection of Algae, Austin, TX, USA) as a biocrust-forming cyanobacterium [25] and *Sporosarcina pasteurii* (ATCC 11859, American Type Culture Collection, Manassas, VA, USA) as a biomineralizing organism [26] (see Tables S1, S2 for culture media). *Nostoc commune* has high tolerance to cold [6] and N-fixing ability [26]. *Sporosarcina pasteurii* has high urease activity and no pathogenicity [12, 27].

We prepared soil with three levels of biomineralization (1, 2, or 3 cycles of biomineralization) plus controls without biomineralization. Plastic Petri dishes (ø 56 mm × 16 mm) were filled with 16 g autoclaved (121 °C, 20 min) silica sand with a particle size of 0.063–0.25 mm. The sand depth was about 5 mm, and the surface was made flat. Biomineralized (MICP-treated) soil was formed by adding *S. pasteurii* (OD_600_ = 1.4) in fresh ammonium–yeast-extract liquid medium (Table S2) and substrate solutions (1 mol/L calcium chloride and 1 mol/L urea) to Petri dishes filled with autoclaved sand. Medium with *S. pasteurii* was added first; then the calcium chloride and urea solutions (1 mL of each) were added at 1-h intervals, drop-wise, evenly across the sand [12, 28]. At 24 h after addition, about 5 mL of sterilized water was poured gently into all the MICP-treated soils to remove the unreacted solution, and the water was removed by micropipette after 1 h, three times. This preparation process was performed one to three times to prepare biomineralized soils with varying degrees of hardening. The number of this repeating process was determined following Wan et al. [29]. We also prepared bare sand without MICP treatment as a control.

The biocrust cyanobacteria were harvested and measured by centrifuge (12 000 × *g*, 15 min) according to Stamatakis and Papageorgiou [30], and 285 mg fresh weight, ~ 3 × that was added by Román et al. [25], was suspended in 3 mL liquid BG-11(-N) medium (Table S1) and added dropwise to each prepared dish, evenly across the surface [28]. We also prepared samples without cyanobacteria (only 3 mL BG-11(-N) medium).

After cyanobacterial inoculation, the samples were cultured in an incubator (LH-241S, Nippon Medical and Chemical Instruments, Osaka, Japan) at 20 ℃ under moderate light intensity with no aeration for 2 weeks. Instead of providing water directly, we placed water-filled vats in the incubator to maintain high humidity. The samples were then stored in a refrigerator (FKG-371F3, Nihon Freezer, Tokyo, Japan) at 7 ℃ for 1 week without light to acclimatize them to the cold environment until the experiment began. We did not add water or moisture during this acclimatizing process. Before conducting the freeze–thaw experiment, we dropped 2.5 mL water into all samples to avoid uneven freezing damage.

To freeze the samples, we stored them in a freezer (GS-1356HC, Nihon Freezer, Tokyo, Japan) at − 14 ℃ (the maximum temperature range that could be set in this freezer) for 14 h without light. We then moved the frozen samples into the refrigerator at 7–8 ℃ for 10 h with room light to thaw the samples. The temperatures and times of the FT cycle were based on Mongolian climatic conditions in spring and autumn. The climatic data was acquired from POWER Data Access Viewer v. 2.0.0 (https://power.larc.nasa.gov/data-access-viewer/) provided by the NASA Langley Research Center (LaRC) POWER Project funded through the NASA Earth Science/Applied Science Program (accessed on 2023/9/23; the summarized data was in Table S3). We selected days with positive maximum and negative minimum temperatures during 2003–2022 and calculated the average of their maximum (7.7 ℃) and minimum (− 5.6 ℃) temperatures. We also calculated the average day length in spring and autumn as 10.2 h. The calculated minimum temperature (− 5.6 ℃), however, could not freeze wet samples completely and uniformly in a preliminary experiment. Therefore, this study employed − 14 ℃ when the freezing process. The freeze–thaw process was performed for 2, 4, or 6 cycles following previous studies [19, 21, 31]. Control samples were stored in the refrigerator at 7–8 °C without light. After the FT cycling, the samples were stored at 7–8 °C without light until measurement. Each Petri plate (degree of hardening by MICP treatment, cyanobacterial inoculation, number of FT cycles) had 3 repetitions, and the total sample number was 96.

### Measured Items

We took all three samples from each of the microcosms and destructively measured indicators of the ecological and physical functions of the biocrust and biomineralized sand: unsaturated hydraulic conductivity (*K*_h_), hardness, thickness, and contents of total C, total N, total carbohydrates, and chlorophyll *a* [28, 32].

*K*_h_ was measured by a Mini Disk infiltrometer (Meter Group, Inc., Pullman, WA, USA). A small amount of sterilized silica sand was added first so as not to break the crust structure; then all samples were dried in a dry heat sterilizer (MOV-112S, Sanyo Electric Co., Ltd., Osaka, Japan) at 50 °C for at least 2 days. After *K*_h_ and infiltration time were measured, the samples were dried again in the sterilizer at 50 °C.

The hardness of each sample was measured as penetration resistance with a pocket penetrometer designed for fruits (KM-1, Fujiwara Scientific Co., Ltd., Tokyo, Japan). The tip of the penetrometer was inserted into the dried soil surface until the surface crust or the biocrusts broke. Each sample was measured three times. The biocrust thickness was measured three times with an electronic caliper. We then crushed and homogenized the soil in each Petri dish for the following measurements.

The contents of total C and total N were measured by NC analyzer (Sumigraph NC-22F, Sumika Chemical Analysis Service Ltd., Japan).

The total carbohydrate content was quantified by the phenol–sulfuric acid method [33] on the basis of the absorbance measured at 488 nm with a spectrophotometer. A reference standard was calculated using d-glucose at different concentrations. If negative values were repeatedly detected in the extraction, the total carbohydrate content was set to 0 in the corresponding sample.

The chlorophyll *a* was extracted in 100% dimethyl sulfoxide (DMSO) and measured by spectrophotometer [34] as absorbance at 665 nm. The content in 5 g of dried sample was determined with the formula shown in Caesar et al. [34]

### Statistical Analysis

To elucidate the effects of biomineralization, cyanobacterial inoculation, and FT cycles on crust properties and ecological functions, we created generalized linear models (Table 1). We selected the level of biomineralization (0, 1, 2, 3), cyanobacterial inoculation (yes or no), the number of FT cycles (0, 2, 4, 6), and their interactions as explanatory variables. We used the above-measured items as response variables. Thickness and chlorophyll *a* were measured only in the cyanobacterial-inoculated samples, so we didn’t include cyanobacterial inoculation as an explanatory variable in the models for these two response variables. We selected the models with the lowest Akaike’s information criterion (AIC) values as the best-fit models [35]. We calculated each explanatory variable’s best-fit line and 95% confidence intervals. We interpreted the explanatory variables in the selected models as significant parameters and the effects as significantly positive or negative if the 95% confidence interval did not cross the zero-effect line [36]. All data were analyzed in R v. 4.3.2 software [37].

**Table 1:** Selected distributions and linkage functions for generalized linear modeling

	Distribution	Linkage function
Log(hydraulic conductivity)	Normal	Identity
Hardness	Normal	Identity
Thickness	Normal	Identity
Total C	Gamma	Identity
Total N	Gamma	Identity
Total carbohydrate + 0.0001	Gamma	Log
Log(chlorophyll *a*)	Normal	Identity

*K*_h_ and chlorophyll *a* were transformed into natural logarithms

## Results

### Effects of Freeze–Thaw Cycles on Physical Properties

Both biocrust inoculation and MICP treatment significantly decreased *K*_h_, but a significant interaction between them weakened their effects (Table 2). Biocrust increased hardness. MICP treatment did not have a significant effect but a significant interaction shows that it enhanced the effect of biocrust inoculation. Biocrust thickness was significantly positively correlated with the level of MICP treatment. There was no effect of FT cycles.

**Table 2:** Model parameter estimates explaining differences in hydraulic conductivity, hardness, and thickness among treatments

	Hydraulic conductivity (*K*_h_)		Hardness		Biocrust thickness	
Parameter	Estimate	95% CI	Estimate	95% CI	Estimate	95% CI
Intercept	**0.0692**	[0.0569, 0.0816]	0.00290	[− 0.00826, 0.0141]	**0.523**	[0.496, 0.549]
BSC	** − 0.0642**	[− 0.0817, − 0.0468]	**0.0257**	[0.00987, 0.0415]	–	–
MICP	** − 0.0215**	[− 0.0281, − 0.0149]	0.00517	[− 8.02e-4, 0.0111]	**0.0234**	[0.00911, 0.0378]
BSC × MICP	**0.0205**	[0.0111, 0.0298]	**0.0184**	[0.00993, 0.0268]	–	–

Models were selected by AIC value. Estimated slopes are in bold if the confidence intervals cross the zero-effect line. *K*_h_ was ln-transformed. The model for thickness was generated from data including only the samples inoculated with biocrusts. "BSC" means the presence or absence of cyanobacterial inoculation and no cyanobacterial inoculation was used as a reference category. The effect of freeze–thaw cycles did not appear

### Effects of Freeze–Thaw Cycles on Chemical Properties

FT cycles had significant but opposite effects on carbohydrate and chlorophyll *a* contents (Table 3). MICP treatment did not have an effect on carbohydrates but had a significantly negative effect on chlorophyll *a*. The interaction between FT cycles and MICP treatment had a significant effect on total carbohydrates, in which MICP treatment weakened the negative effect of FT cycles.

**Table 3:** Model parameter estimates explaining differences in total carbohydrates and chlorophyll *a* among treatments

	Total carbohydrate		Chlorophyll *a*	
Parameter	Estimate	95% CI	Estimate	95% CI
Intercept	**3.269**	[2.625, 4.019]	**1.06**	[0.654, 1.46]
FT cycles	** − 0.373**	[− 0.566, − 0.182]	**0.141**	[0.0563, 0.225]
MICP	− 0.207	[− 0.600, 0.179]	** − 0.304**	[− 0.473, − 0.135]
FT cycle × MICP	**0.156**	[0.0480, 0.265]	–	–

Models were selected by AIC value. Estimated slopes are in bold if the confidence intervals cross the zero-effect line. Both models were generated from data including only the samples inoculated with biocrusts

Both treatments significantly increased the total C content, but there were no effects of FT cycles or any interaction terms on it (Table 4). Both treatments also significantly increased the total N content, and FT cycles significantly decreased it. The best model for N content had no interaction terms.

**Table 4:** Model parameter estimates explaining differences in total C and total N among treatments

	Total C		Total N	
Parameter	Estimate	95% CI	Estimate	95% CI
Intercept	**0.00798**	[0.00749, 0.00851]	**0.00236**	[0.00182, 0.00304]
BSC	**0.0389**	[0.0364, 0.0416]	**0.00624**	[0.00507, 0.00762]
FT cycles	–	–	** − 1.87*e* − 4**	[− 3.31*e* − 4, − 4.57*e* − 5]
MICP	**0.0432**	[0.0417, 0.0448]	**0.0110**	[0.0101, 0.0120]

Models were selected by AIC value. Estimated slopes are in bold if the confidence intervals cross the zero-effect line. "BSC" means the presence or absence of cyanobacterial inoculation and no cyanobacterial inoculation was used as a reference category.  Best models did not include any interaction terms

## Discussion

### Response of Biocrust and MICP-Treated Sand to FT Cycles

Our experiment focused on the effect of repeated FT cycles on the structure and development of cyanobacterial biocrust on MICP-treated soil. The simulated FT cycles did not affect the physical structure or related functions but appeared to change the productivity and nutrient condition of the crust.

FT cycles had no effect on the physical structure or hydraulic conductivity of the cyanobacterial biocrust on the MICP-treated soil (Table 2). Zhao et al. [38] stated that repeated FT cycles could alter the microstructure of the soil and decrease water infiltration, but other studies showed that the soil microstructure remained unchanged after 5–15 FT cycles [39–41]. We based the FT conditions on the Mongolian climate (Table S3), using up to six cycles of − 14 ℃ for 14 h and 7–8 ℃ for 10 h. In agreement with previous studies (e.g., [39]), this number of FT cycles was enough to show the structural robustness of the biocrust on MICP-treated sand against the major FT stress during autumn and spring.

It should be noted that our study focused on the topsoil response to FT cycles. Wet underneath soil can modify the biocrust community and physical structure in the soil surface, like frost heaving [2] and deformation caused by the FT phenomenon [42]. On the other hand, both cyanobacterial inoculation and biomineralizing treatment significantly decreased the hydraulic conductivity (the easiness of water infiltration) in our study (Table 1, also see the “Synergy and Conflict Between Biocrust and MICP-Treated Sand Under FT Cycles” section). This result suggests that biocrust and biomineralized layers could restrict water infiltration into the underlying soil and minimize the frost heaving and underneath soil expansion caused by the FT cycles. The mechanism involving the soil layer can be clarified, for example, by the experiment cultivating the samples in columns instead of the Petri dishes (e.g., [42]).

FT cycles significantly changed the contents of total carbohydrates and total N (Tables 3 and 4). The decrease in total N content by FT cycles was reported in other studies [21]. Denitrification caused by FT cycles could explain this N loss [43], as FT cycles can break the soil microbial cells and leach the nutrients and enzymes from them [44], accelerating N loss.

FT cycles also significantly decreased total carbohydrates. Most carbohydrates produced by cyanobacteria are exopolysaccharides (EPS) [45]. EPS enables cyanobacteria to adapt to repeated FT cycles [46], and therefore, we hypothesized the positive correlation between the carbohydrate amount and the number of FT cycles. Our result, however, means a decrease in total carbohydrate including EPS by the repeated FT cycles. The result could be explained by the activity of enzymes, such as glycolytic enzymes, released from the cells collapsed by the FT cycles. Although low temperatures enough to freeze have already been reported to increase EPS production in marine bacteria [47], our study could confirm the relationship between cyanobacterial carbohydrate production and the FT phenomenon. The molecular weight detection [28] might show the direct relationship between EPS amount and the FT cycles.

FT cycles significantly increased chlorophyll *a* in the biocrust. This increase indicates increasing biomass [48], which means that the FT cycles facilitated biocrust development during our experiment. A possible explanation for this result is that our simulated FT cycle might be mild for *N. commune*, which survived and grew during 45 soil temperature changes from − 12 to + 26 °C [6]. Although several candidate species for artificial cyanobacterial biocrust have been summarized [1], our study confirms the tolerance of inoculated *N. commune* to mild FT cycling and indicates *N. commune* is a suitable candidate for land restoration in northeast Asia.

### Synergy and Conflict Between Biocrust and MICP-Treated Sand Under FT Cycles

Our other goal was to test the interaction between cyanobacterial inoculation and the MICP-treated sand under FT cycling. Our results show both a negative effect of MICP treatment on the biocrust organism and beneficial and protective effects on structural aspects of the biocrust.

Thickness and hardness are structural development indicators of biocrust [48], and both showed increasing trends with the MICP-reacting level (Table 2). MICP treatment and biocrust inoculation significantly decreased *K*_h_ (Table 2), indicating a reduction in soil porosity [38]. MICP treatment of sand particles accretes nearby particles to create larger aggregated particles [14]. The microbes can start MICP reactions when there are substrates surrounding them and form cell aggregates enclosed within CaCO_3_ that they secrete [49]. The biocrusts developing on biomineralized soil might involve non-CaCO_3_-coated sand particles, CaCO_3_-coated ones, and CaCO_3_-secreted cells without sand particles and thus form a dense and thick structure of the surface crust. This mechanism could explain the trends in *K*_h_, hardness, and biocrust thickness, suggesting that biomineralization would support the physical development of the biocrust.

The increase in total C from the MICP reaction can be explained by the CaCO_3_ precipitated through biomineralization. Organic material in the *S. pasteurii* cells might increase total N with the level of MICP reactions in the precipitated minerals. The microbes become sealed within the precipitated CaCO_3_ attached to the sand grains [14, 49] and therefore are not washed away by rinsing.

Chlorophyll *a* and carbohydrate contents are indexes of biocrust development [1, 48]. They were not, however, increased by the MICP treatment (Table 3). These decreasing trends could be explained by the adsorption of metal ions in the soil by CaCO_3_ [50]: the precipitated CaCO_3_ might deprive the soil of essential metal ions (e.g., Mg^2+^), inhibiting cyanobacterial growth and bioactivity, such as carbon fixation and soil nutrient improvement. The amount of this adsorption, however, seems to depend on the surface area of CaCO_3_ crystals [50]. Therefore, the addition of plenty of medium before cyanobacterial inoculation might saturate the CaCO_3_ surface and moderate the negative impacts of biomineralization.

The same conflict was observed also in *K*_h_: both biocrust inoculation alone and MICP treatment alone decreased *K*_h_ significantly, but the interaction term had a significant positive effect (Table 2). Cyanobacteria often digest minerals from stones [51], but the inoculated *N. commune* did not dissolve CaCO_3_ (preliminary test) or decrease total C content (Table 4). One possible explanation, except for digesting, is microstructural changes in the combined crust: the biocrust-forming cyanobacteria spread filaments in all directions and capture the surrounding particles [52]; they might penetrate the biomineralized layer, and the resultant filaments or cracks in that layer might function as water capillaries. Although our experiment did not test this hypothesis, microscopic observations might reveal the reason for this conflicting effect on *K*_h_.

MICP treatment itself showed an insignificant reducing trend in the total carbohydrate content (Table 3). It is noteworthy that it seemed to moderate the negative effect of FT cycles on the total carbohydrate content, as indicated by the significant positive interaction between MICP treatment and FT cycles (Table 3). Carbohydrate production in cyanobacteria is regulated by several environmental factors, including temperature [53]. MICP-treated soil has high heat conductivity [54] and might make it easier for the cyanobacteria to detect the temperature change and to arrange their bioactivity including carbohydrate production. Our study could not detect how MICP treatment soil protected the total carbohydrate content, but this protective effect would prevent the degradation of the inoculated biocrust structure from temperature changes in field conditions.

## Conclusion

The cyanobacterial biocrust on biomineralized soils was able to maintain its physical structure and related ecological functions under at least moderate FT cycles. The results suggest that the MICP reaction can enhance the physical structure of the cyanobacterial biocrust and protect carbohydrates from degradation by FT cycles, although it did not enhance biocrust growth indicators. Our experiment focused on the topsoil layer and eliminated the influence from the underlayer soil but biocrust with biomineralized soil might endure FT cycling during spring and autumn, contributing to degraded land restoration in cold arid regions with its structural robustness.

### Supplementary Information

Below is the link to the electronic supplementary material.

Supplementary file1 (DOCX 20 KB)

## Data Availability

The climatic data is available at POWER Data Access Viewer v. 2.0.0 (https://power.larc.nasa.gov/data-access-viewer/). The summarized data was also available in supplementary information. The data measured in the current study are available from the corresponding author on reasonable request.

## Code Availability

The R code used for analyses is also available from the corresponding author on reasonable request.
