# Supplementary material for: Cyanobacterial Biocrust on Biomineralized Soil Mitigates Freeze–Thaw Effects and Preserves Structure and Ecological Functions
Source: Microb Ecol. 2024 May 10;87:69. doi: 10.1007/s00248-024-02389-w (PMC11087357; doi:10.1007/s00248-024-02389-w)
Supplement: Supplementary file 1 [file 248_2024_2389_MOESM1_ESM.docx]

Cyanobacterial biocrust on biomineralized soil mitigates freeze–thaw effects and preserves structure and ecological functions

Keiichi KIMURA*, Toshiya OKURO

Department of Ecosystem Studies, Graduate school of Agricultural and Life Sciences, The University of Tokyo, 1-1-1 Yayoi, Bunkyou-ku, Tokyo, 113-8657, Japan

*Contact: keiichi-kimura045@g.ecc.u-tokyo.ac.jp

Microbial Ecology

# Supplementary information

Table S1 Chemical composition of BG 11(-N) and trace metal solution. The receipt is based on UTEX (accessed on 2023/12/20).

| **Medium / solution** | **Component** | **Final concentration** |
| --- | --- | --- |
| BG-11 (-N) | K_2_HPO_4_ (Sigma P 3786) | 0.22 mM |
|  | MgSO_4_•7H_2_O (Sigma 230391) | 0.3 mM |
|  | CaCl_2_•2H_2_O (Sigma C-3881) | 0.24 mM |
|  | Citric acid•H_2_O (Fisher A 104) | 0.012 mM |
|  | Ferric ammonium citrate | 0.02 mM |
|  | Na_2_EDTA•2H_2_O (Sigma ED255) | 0.002 mM |
|  | Na_2_CO_3_ (Baker 3604) | 0.18 mM |
|  | BG-11 trace metals solution | (add 1 mL/L) |
| BG-11 Trace Metals Solution | H_3_BO_3_ (Baker 0084) | 46 mM |
|  | MnCl_2_**•**4H_2_O (Baker 2540) | 9 mM |
|  | ZnSO_4_**•**7H_2_O (Sigma Z 0251) | 0.77 mM |
|  | Na_2_MoO_4_**•**2H_2_O (J.T. Baker 3764) | 1.6 mM |
|  | CuSO_4_**•**5H_2_O (MCIB 3M11) | 0.3 mM |
|  | Co(NO_3_)_2_**•**6H_2_O (ACROS 10026-22-9) | 0.17 mM |

Table S2 Chemical composition of ammonium–yeast extract medium (ATCC 1376).

| Component | Amount |
| --- | --- |
| Yeast extract | 20 g |
| (NH_4_)_2_SO_4_ | 10 g |
| 0.13 M Tris buffer (pH 9.0) | 1 L |
| Agar (if need) | 20 g |

Note: Autoclave ingredients separately. No growth occurs when ingredients are sterilized together.

Table S3 Average maximum and minimum temperatures on days when the freeze–thaw phenomenon must occur. Spring is January to April; autumn is September to December.

| Season | Spring | | | Autumn | | |
| --- | --- | --- | --- | --- | --- | --- |
| Year | Day | Max. temp.^†^ | Min. temp. ^†^ | Day | Max. temp. ^†^ | Min. temp. ^†^ |
| 2013 | 43 | 7.01 | −7.76 | 46 | 7.95 | −4.72 |
| 2014 | 42 | 8.87 | −5.64 | 46 | 8.66 | −4.71 |
| 2015 | 38 | 7.84 | −6.62 | 38 | 8.37 | −4.69 |
| 2016 | 49 | 8.64 | −5.13 | 37 | 7.07 | −5.29 |
| 2017 | 54 | 7.42 | −6.53 | 43 | 7.35 | −5.10 |
| 2018 | 49 | 9.42 | −5.28 | 54 | 8.18 | −4.15 |
| 2019 | 51 | 7.86 | −6.21 | 42 | 7.16 | −4.61 |
| 2020 | 50 | 8.08 | −6.19 | 47 | 6.77 | −4.83 |
| 2021 | 59 | 7.80 | −6.64 | 50 | 7.00 | −4.97 |
| 2022 | 48 | 7.10 | −6.87 | 50 | 5.85 | −5.79 |
| Average | 48.3 | 8.01 | −6.29 | 45.3 | 7.44 | −4.89 |

†Air temperature. Data obtained from POWER Data Access Viewer (v. 2.0.0, NASA, accessed 2023/9/23).
